# A Modification of Heller’s Test

**Published:** 1901-12

**Authors:** 


### Abstracts and Selections.

### A Modification of Heller's Test.

In the November 2nd number of *American Medicine,* Dr. C. F. M. Leidy describes a modification of Heller's contact test which he claims reduces the amount of reagent necessary and renders the result more accurate.

He immerses a glass tube of small interior diameter in the urine to a depth of about two inches, then closing the tube by pressing a finger over the free end he withdraws it, dries the lower end, and then slowly lowers it into nitric acid until the acid and urine are about equal in depth. This gives a sharp line of contact, which will not be disturbed by the agitation incident to ordinary handling. The test can be carried out by using an ordinary straight medicine dropper. R.

